# Exploring Potential Obstacles and Solutions to Implementing a Safety Plan Through a Brief Three-Week Safety Planning Group Intervention: Findings over Nine Months

**DOI:** 10.3390/healthcare14162521

**Published:** 2026-08-13

**Authors:** Suzanna Chalecka, Anna Glynn, John Bogue, Ruth Melia, Mary O’Sullivan, Karen Young, Jim Duggan, Hannah Wood, Yvonne Bergmans, Shane McInerney

**Affiliations:** 1 School of Psychology, University of Galway, H91 W5P7 Galway, Ireland; 2 Health Service Executive West and Northwest, H91 N973 Galway, Ireland; anna.glynn0@proton.me (A.G.);; 3 Psychology Department, University of Limerick, V94 T9PX Limerick, Ireland; 4 Resource Office for Suicide Prevention, Health Service Executive West and North West, H91E342 Galway, Ireland; mary.osullivan@hse.ie; 5 School of Computer Science, University of Galway, H91 W5P7 Galway, Ireland; 6 Department of Psychiatry, Temerty Faculty of Medicine, University of Toronto, Toronto, ON M5T 3M7, Canada; 7 Department of Psychiatry, University of Galway, H91 W5P7 Galway, Ireland

**Keywords:** safety planning, suicide, group, intervention, obstacles, solutions

## Abstract

**Highlights:**

**What are the main findings?**

- Two themes described the practical use of safety plans: External Obstacles and Solutions (environmental and situational barriers, interpersonal barriers to support, behavioural and contextual influences, adaptive coping and support strategies) and Internal Obstacles and Solutions (executive functioning and motivation, emotional distress and self-sabotage, stigma and help-seeking, and personal resilience and future-oriented coping).
- The group context facilitated the identification of both obstacles and corresponding solutions, demonstrating how shared reflection can support individuals in anticipating and responding to barriers of safety plan use.

**What are the implications of the main findings?**

- Safety Plan use is shaped by both internal and external factors that extend beyond the plan itself.
- Tailored supports that address the barriers identified in this paper may improve the practical use of safety plans for people experiencing suicidality.

**Abstract:**

**Background/Objectives:** Safety Planning Group Interventions (SPGI) represent a novel approach to suicide prevention. Following this three-week intervention, an individualised safety plan was produced by each participant. Within the context of the group setting, obstacles and solutions that may promote engagement with safety plans were discussed. Examining the obstacles and solutions identified by group participants may reveal how to better support people experiencing suicidality and what supports are identified that may support safety plan implementation. This research sought to determine what obstacles participants face when implementing their safety plans and what solutions could be identified for these obstacles. **Methods:** The study used a qualitative research design. Fifty participants completed an in-group list of potential obstacles and solutions to implementing safety plans, which were analysed using Reflexive Thematic Analysis. **Results:** The analysis yielded the results for two themes. The first theme, External Obstacles and Solutions, with subthemes of environmental and situational barriers, Interpersonal barriers to support, Behavioural and contextual influences, Adaptive coping and support strategies, was identified. The second theme, Internal Obstacles and Solutions, with subthemes of executive functioning and motivation, emotional distress and self-sabotage, stigma and help-seeking, and personal resilience and future-oriented coping, was identified. **Conclusions:** The findings of this research illustrate the various obstacles and their corresponding solutions that may facilitate safety plan implementation. Recommendations for future research are discussed.

## 1. Introduction

Suicide remains the leading cause of death worldwide and continues to be a serious public health concern [1]. In Ireland, in 2022, there were 500 recorded deaths of people who died by suicide [2]. Research has indicated that with timely, evidence-based, and low-cost interventions, the high number of people who die by suicide can be prevented [1]. Suicidality refers to suicidal thoughts, suicidal attempts, suicidal ideation, and suicidal behaviours, covering a broad spectrum of suicidal risk and varying levels of emotional suffering and intent [3].

Safety Planning Interventions (SPIs) have been shown to reduce suicide deaths and suicide-related behaviours [4,5]. SPIs are becoming more frequently administered and have been gaining popularity in both clinical and community settings [5,6]. There are typically six components involved in SPIs, which are aimed at reducing suicidality through the development of personalised lists of coping and personal support strategies to be used ahead of a suicidal crisis [7]. These include becoming aware of and recognising personal early warning signs, identifying internal coping strategies, turning to self-identified means of social support to distract from suicidal thoughts, contacting members of your social support network ahead of a crisis, contacting professionals, and reducing the use of lethal means and methods [5]. A key aspect of safety planning is collaboration by co-creation. SPIs focus on keeping the individual at the centre of the planning process, encouraging them to help develop practical and meaningful strategies they feel comfortable trying [6]. Furthermore, along with traditional paper-based safety planning, safety plans have also been adapted into digital formats [8,9]. While most of the research has focused on the veteran population, research has shown that SPIs can be applied across a wide range of populations and settings [10]. Systematic reviews examining the effectiveness of SPIs show that they can lead to improvements in lowering the risk of suicidality. Evidence from systematic reviews and meta-analyses indicates that SPIs reduce suicidal ideation and behaviour and improve treatment engagement [6,10,11]. However, recent reviews highlight that interventions vary significantly in intervention delivery and note that little research has examined how individuals actually use safety plans in practice or what prevents implementation [11]. Although SPIs have been demonstrated to be effective, their benefits depend on individuals being able to implement the strategies detailed within their safety plan during periods of distress. Barriers to implementation remain poorly understood.

Participants with lived experience of mental health difficulties can face various challenges and obstacles to treatment adherence. Adherence and non-adherence to treatment for mental health problems are multifaceted phenomena that are influenced by a person’s environment and context, with both social and institutional factors having been found to support treatment adherence [12,13,14]. Treatment adherence among adults experiencing suicidality is influenced by multiple factors. A systematic review found that logistical barriers, stigma, and negative treatment experiences commonly impede engagement, whereas therapeutic alliance and collaborative, empowering interventions facilitate treatment continuation [13]. Moreover, continuity of care appears to play an important role in suicide prevention. Greater adherence to a structured follow-up programme after a suicide attempt has been associated with lower rates of repeat suicidal behaviour and hospital admissions [15]. Treatment adherence and safety plan implementation both require individuals to engage with therapeutic strategies during periods of distress. Consequently, barriers influencing treatment engagement may also affect individuals’ ability to implement their safety plans. It is therefore important that interventions, such as the one described in this paper, be administered as they seek to identify not only the obstacles that participants face in implementing their safety plans but also explore solutions to these obstacles within the intervention setting, by means of a collaborative solution-seeking approach.

Modern perspectives acknowledge the vital role of lived and living experience in suicide prevention research. Regrettably, however, such experiences have been minimally integrated into the development of existing suicide prevention interventions. There is also limited guidance available on how to incorporate participants with lived experience into the development of such interventions [16]. A recent scoping review found inconsistencies in how such involvement is defined, implemented, and reported, highlighting the need for more transparent and meaningful co-design approaches in intervention development [17]. Incorporating these insights is crucial in ensuring that prevention strategies effectively address the very needs of those they are designed to support [5,17,18]. Additionally, a deeper understanding of individuals’ experiences with the SPI is necessary to inform both clinical practice and future research, ultimately improving SPI effectiveness and reducing suicide and related distress [5]. To our knowledge, there is no research examining the obstacles to safety plan implementation, except for research examining the obstacles to safety planning for suicidality for autistic individuals. The results of this study suggest that clinical adaptations and a patient-oriented approach supported participants to engage in the safety planning intervention [19]. Qualitative research suggests that young people with lived and living experience of suicide value meaningful involvement in suicide research when supported, highlighting the importance of trauma-informed and participant-centred research [20]. Although qualitative research has explored experiences of using SPIs [5], little research has specifically examined the obstacles individuals encounter when attempting to implement their safety plans or the solutions they identify to overcome challenges to safety plan use in times of distress. Existing work has largely focused on intervention effectiveness rather than implementation, and lived experience perspectives have remained underrepresented. Addressing these gaps may improve the acceptability, feasibility, and real-world implementation of SPIs.

This study explores the obstacles and solutions that may enhance the practical use of safety plans as identified by participants of a three-week SPGI delivered over the course of nine months. It aims to contribute to the growing body of research supporting SPGIs for individuals experiencing suicidality. Specifically, the study explores obstacles to following a safety plan and the corresponding solutions, to foster discussions that promote a climate of safety among people who are experiencing suicidality.

### Aims

This study aimed to explore the obstacles encountered by participants when implementing safety plans and to identify potential solutions to address these challenges.

## 2. Materials and Methods

### 2.1. Participants

The sample consisted of 50 participants attending inpatient or outpatient mental health services in the west of Ireland who had recently experienced suicidality. For referral, suicidality was defined as recent suicidal ideation, a recent suicide attempt, or recent self-harm. Participants therefore represented a range of clinical presentations, including those experiencing suicidal thoughts, suicide-related behaviours, and/or recent suicide attempts. A recent suicide attempt was not an inclusion criterion. Participants were referred by their treating clinicians based on clinical judgement that they would benefit from the intervention. Referrals could be made by any member of the mental health team and were based on clinical judgement, provided that participants met the study’s established inclusion and exclusion criteria. Individuals who had experienced recent suicidality were considered eligible for referral if they satisfied the study eligibility criteria. Following referral, the SPGI research assistant contacted participants by telephone and sent a letter of invitation, inviting them to attend an initial assessment appointment. During this appointment, written informed consent was obtained, demographic information was collected, and baseline psychometric measures were administered. Exclusion criteria for participation included: involuntary admission to the hospital (if inpatient at the time of referral), an inability to communicate effectively in English, or an active psychotic or dementia-related illness. Substance misuse was not an exclusion criterion. The standard protocol for completing this SPGI was the completion of a battery of self-assessment psychometric measures before and after the three-week intervention. However, the data collected and used for this research paper were collected during Session 3 of the intervention. Therefore, the number of participants reported in this paper reflects all participants who attended Session 3 of the intervention (N = 50) over the nine months, including those who did not complete the post-group psychometric measures. For participant demographics, see Table 1.

### 2.2. Treatment Protocol

Over a period of nine months, participants were invited to attend the SPGI. Before the intervention, participants met with the research assistant and completed a set of psychometric assessments. At this time, participants were allowed to ask any questions or raise concerns about the intervention. Written, informed, and freely given consent was obtained at this time. The intervention took place one day a week over three consecutive weeks, with each session lasting approximately 90 min. Following the intervention, participants completed the same set of psychometric measures and gave additional feedback on their experience of the intervention. The treatment material was adapted from the 20-week Skills for Safer Living Program [21] and was delivered by a team of professionals from various disciplines, including Occupational Therapy, Psychiatry, and Psychology. Facilitators of the intervention completed training under Dr Bergmans, as well as a two-day Skills Training for Suicide Prevention and Self-Harm Mitigation [22].

Before safety plan development, participants received psychoeducation on suicide and safety planning, and were taught core skills of grounding strategies, recognising safety and risk, identifying community networks, recognising emotions, understanding early warning signs, and the physical signs of stress. During the third and final session of this intervention, each participant completed an individualised safety plan. It was hoped that with assistance from facilitators, participants would create a safety plan individually tailored to their needs and circumstances, which reflected the skills and concepts introduced throughout the intervention and made use of resources within the local community. Participants were also encouraged to create copies, either by photocopying their safety plan or accessing the SafePlan app [8,9]. The final step of the intervention involved a discussion of potential obstacles and corresponding solutions related to implementing the safety plan. Participants, in collaboration with facilitators, identified barriers that could personally hinder safety plan implementation. Subsequently, using the same approach, participants explored potential solutions to address the identified challenges. This list was compiled over the nine-month intervention period.

### 2.3. Data Collection

Participant responses were collected during the final session of the intervention, during which a discussion focused on identifying potential obstacles to the practical use of safety plans and their corresponding solutions. Throughout each discussion, participant responses were documented on a whiteboard visible to the group. Responses were recorded as key phrases and documented group outputs reflecting participants’ contributions and were organised according to the identified obstacle and its corresponding solution.

Following each session, photographs of the completed whiteboard were taken to ensure accurate documentation of the data generated during the discussion. These images were reviewed alongside researcher notes and used to compile a structured list of obstacles and solutions for each group. Data from all groups were subsequently collated into a single dataset for analysis. Responses generated by each group were independently collected, and no materials from previous groups were presented to subsequent groups. The final dataset consisted of participant-identified and discussed obstacles and solutions, which were analysed using reflexive thematic analysis to identify patterns of shared meaning across the data. Although participant responses were recorded as statements rather than verbatim transcripts, the objective of the analysis was not to quantify responses but to develop themes representing shared patterns of meaning across participants, the obstacles and solutions described by participants. Reflexive thematic analysis was selected because it provides a flexible approach to identifying patterns of shared meaning across qualitative datasets of varying forms [23].

### 2.4. Qualitative Analysis

Verbal obstacles and solutions from the intervention participants were collected and compiled into a comprehensive list following the final month of the intervention. Data were collected across multiple group sessions, with each group discussing the end of the final session obstacles and solutions to safety plan use. Participants were not provided with responses or themes created by previous groups. Following data collection, responses from all groups were collated and analysed using reflexive thematic analysis to identify patterns of shared meaning across the dataset [23]. The participant sample reflects the total number of participants who attended the group sessions; however, the analysis consisted of the collective dataset which had been generated across groups rather than frequency and/or endorsement of specific items. Therefore, findings represent themes identified across participant discussions and don’t indicate the proportion of participants endorsing each theme.

During each group discussion, participant responses were recorded on a whiteboard visible to all participants by a facilitator. While recording the discussion points on the whiteboard, facilitators checked in with the group about what was being captured to ensure that it accurately reflected participants’ intended meaning and invited clarification where required. Photographs of the whiteboards were taken after each session, and the material was transferred to a document with outputs from all sessions. Any additional participant contributions or relevant discussion points were also included here. This process allowed for a clear and accurate record of participant perspectives. Verbatim transcripts were not collected for this study; the analysis was not intended to capture individual narratives. The focus was on identifying patterns across collectively generated group discussions. While this limits the availability of direct quotations, meaning was supported through the process described above.

Themes were developed through iterative engagement with the data, as similar obstacles and consecutive solutions were identified by various participant groups. The data were analysed in accordance with Braun and Clarke’s Reflexive Thematic Analysis (RTA) [24]. This method allowed for the data to be analysed under the view that qualitative research is subjective, reflexive, and creative [23,24]. As RTA does not follow the sometimes-rigid forms of TA through being research-driven, flexible, and interpretive, it naturally appeared to be the most suitable form of analysis for the dataset generated in this study [23,24]. The primary author familiarised themselves with the dataset through repeated reading of the participant responses. Initial codes were generated from the data, with codes assigned to meaningful segments of text that described participants’ perceived obstacles and proposed solutions for implementing safety plans. Coding was repeated and refined, allowing codes to be reorganised as analysis continued. Codes which reflected similar patterns of meaning were grouped to generate preliminary themes. These themes were reviewed and refined through repeated engagement with the dataset to ensure they accurately represented participants’ accounts and were distinct from one another. Theme names and definitions were developed through discussion and refinement. A contextualist approach was adopted, recognising that participants’ experiences reflect both personal realities and the broader social, relational, and environmental contexts in which they occur.

### 2.5. Reflexivity Statement

The primary author was a research assistant involved in the wider intervention project and was present during the delivery of the intervention and therefore brought familiarity with the group context to the analysis. In this role, the researcher carried out pre- and post-intervention assessments with participants and attended all group sessions. This resulted in familiarity with participants, the intervention context, and the experiences discussed during sessions. Consistent with the principles of Reflexive Thematic Analysis, this positionality was viewed as a resource that informed interpretation while also requiring ongoing critical reflection [23,24]. Throughout the analytic process, the researcher was aware of how prior knowledge and assumptions could shape interpretation and engaged in discussions with members of the research team during theme development.

The wider research team also had extensive experience in suicide prevention research and clinical practice. This level of involvement provided valuable contextual understanding of the intervention and participant experiences, but also had the potential to influence data interpretation and theme development. In keeping with a reflexive thematic analysis approach, the primary author engaged in reflexive discussions with a member of the research team regarding coding and theme development. These discussions supported reflection on interpretations and helped identify any influences arising from the researcher’s involvement with the intervention and participants.

## 3. Results

The analysis generated two themes: External Obstacles and Solutions and Internal Obstacles and Solutions.

### 3.1. Theme One–External Obstacles and Solutions

The first theme encompassed challenges that participants struggled to manage due to external circumstances. Several subthemes were identified in this category, such as time of day, triggers, people identified as being part of the participants’ support network being unavailable, negative interpersonal relationships, distressing environments, work/academic stress, substance misuse, social media, and the impact of others ending their lives see Table 2. Rather than presenting as isolated barriers, these challenges often interfered with multiple stages of the SPI, particularly with recognising warning signs, using internal coping strategies, accessing social support, and reducing access to means.

Obstacles that interfered with SPI steps, specifically the first steps involved in SPI, were particularly prominent. Participants often described feeling more vulnerable to distress at certain times or days of the week. This may have been linked to patterns of isolation or a routine that no longer served them. To mitigate these effects, implementing coping strategies such as setting calendar reminders, rewards for engaging in positive behaviours, and planning enjoyable activities or events was suggested. These coping strategies, in line with early steps of SPI, may be more accessible.

Triggers and certain distressing environments, which may have interfered with the participants’ ability to recognise their warning signs and implement identified coping strategies, were also discussed. These triggers were described as causing emotional spirals, while certain locations were associated with past trauma, anxiety, or other negative experiences. Participants discussed solutions to these by making their coping strategies more adaptable and dependent on the context in which they were experienced, including changing walking routes or environments, identifying safe spaces, and using sensory grounding techniques such as cold-water therapy, eating spicy foods, and various grounding exercises. These solutions appeared to strengthen safety planning by providing options that could be used immediately when participants were in distress.

Challenges in social support emerged as one of the most significant obstacles to safety plan use during times of distress. Participants often discussed situations in which their identified support person was unavailable, leaving them feeling isolated and unsure how to proceed with components of their safety plan. This obstacle appears to be particularly influential as it interferes with more than one step on a safety plan, this being contacting people identified in your support network and also reaching out to family members or professionals. As a solution, participants described expanding their support network, identifying alternative contacts, practising self-care, and engaging in their comfort activities when their identified support was unavailable. Some also found it helpful to examine situations from an external/more objective perspective to reduce feelings of hopelessness. Participants spoke about the importance of identifying people to avoid during times of distress, as a preventative and protective measure. Participants reported that relationships with certain people may have at times led to increased distress, which made it harder to identify the need to use their safety plan. To protect against this, participants discussed setting boundaries, limiting contact, creating buffers, and identifying people to avoid during periods where they may have felt more vulnerable. This worked as a preventative adaptation to aspects of SPI which identify social supports, helping participants distinguish between relationships that promoted safety and those that increased risk. Participants also expressed distress following the loss of someone to suicide. This experience was often accompanied by grief, confusion, and emotional overwhelm. To maintain safety and cope with these feelings, individuals highlighted the importance of relying on social support networks and acknowledging grief. This suggests that bereavement could temporarily disrupt several SPI steps simultaneously, requiring additional flexibility in how a safety plan is used.

Work or Academic-related stress was discussed as a significant obstacle. Here, the importance of identifying familiar and safe spaces was once again discussed as a potential solution. Other strategies involved regular self-check-ins, maintaining a functional work–life balance, and seeking support when needed. Other solutions included budgeting to reduce financial pressure and prioritising basic needs. These strategies appeared to support both the recognition of warning signs and the implementation of coping strategies during stressful times. Substance use was identified as both a coping mechanism and a potential obstacle. Participants who struggled with substance misuse highlighted the importance of seeking support groups (e.g., Alcoholics Anonymous), talking to someone who understands their experiences, and gradually reducing or limiting substance use where possible. Some also emphasised the power of visualising a positive future and giving excess substances to family or friends as a safeguard. Lastly, participants described social media as an obstacle to maintaining their safety. Participants reported feeling overwhelmed by negative content, comparisons, and news overconsumption (‘doom scrolling’). As a potential solution, participants discussed unfollowing certain accounts, limiting time spent on apps, and avoiding doom scrolling. Overall, external obstacles rarely seemed to prevent safety plan use entirely; they often required participants to adapt how specific SPI steps were carried out.

### 3.2. Theme Two–Internal Obstacles and Solutions

The second theme discussed the internal psychological barriers that made it difficult for participants to engage with their safety plans. It included challenges such as executive functioning, lack of motivation, emotional intensity, self-sabotage, and shame related to mental health difficulties, see Table 3. Unlike the external barriers described above, these obstacles often interfered with participants’ ability to enact the safety plan even when support was available. Participants described adapting several SPI steps, particularly using internal coping strategies and seeking support, to overcome these internal obstacles. Across the theme, identifying personal reasons for living emerged as one of the most important reasons for continued use of a safety plan (Table 4).

Participants identified executive functioning difficulties as a significant obstacle to maintaining well-being and safety plan implementation. Challenges included struggling with planning, progressing toward self-identified goals, and completing tasks. These difficulties often disrupted the implementation of the SPI steps, even when participants recognised that they were becoming more distressed. To address these challenges, participants discussed the importance of finding or reminding themselves of their reasons for living, setting phone reminders, making photocopies of their safety plans, and using resources like the SafePlan app [8,9]. Participants also mentioned planning for distressing times, holding oneself accountable and sharing safety plans with people within their safety network. Participants also found it helpful to remember what had worked for them in the past during times of distress and remind themselves that ‘this will pass’. These changes appeared to reduce the cognitive demands of implementing the safety plan during periods of heightened distress.

Participants spoke of struggling with motivation, resulting in difficulty in engaging in self-care. To address this, participants discussed the importance of seeking support rather than trying to overcome challenges alone. Participants discussed that reminding themselves of their reasons for living, such as loved ones, wishes, and future goals, helped maintain motivation. An approach of “try, try, and try!” was discussed, acknowledging that if one thing does not work or if one person is not available, move on and try the next. This suggests that reasons for living acted not simply as one component of the SPI but perhaps as a resource that supported engagement across multiple stages of the SPI process.

Self-Sabotage and negative thought patterns were often discussed as a significant challenge. This included not seeking help and withdrawing from support networks. To aid with this, participants emphasised the importance of self- awareness and recognising harmful behaviours they may be engaging in. These patterns often prevented participants from moving to the interpersonal components of the SPI despite recognising their distress. Participants found that reminding themselves of their reasons for living and acknowledging that their loved ones cared for them helped with a negative self-sabotaging mindset. Additional solutions included preparing for difficult times in advance, using positive affirmations, and reminders from loved ones during times of distress. Writing letters to their future selves, outlining reasons for living, and texting a friend for support were also mentioned as helpful resources. These strategies appeared to reduce the impact of self-sabotaging thoughts by making help-seeking more achievable.

Finally, participants described shame surrounding their mental health difficulties as an obstacle to self-compassion and help-seeking. Feelings of embarrassment or fear of judgment often resulted in participants hiding their distress from others or avoiding contact with support persons, thereby disrupting the social support components of the SPI. To overcome this, participants emphasised contacting people who made them feel safe and not judged. Looking at photographs of loved ones, identifying people in their network who would respond without criticism, and spending time in environments where they felt safe helped reduce feelings of shame and increased their willingness to seek support when needed.

Overall, internal obstacles seemed to mostly affect the participants’ capacity to engage with their safety plans rather than their knowledge of what to do in times of heightened distress. Among these, executive functioning difficulties, reduced motivation, and self-sabotaging thoughts seem to be of particular importance as they prevented participants from engaging with SPI steps. Participants responded by adapting to reduce decision-making effort, increase accessibility, strengthen motivational reminders, and facilitate help-seeking. Across all subthemes, identifying with reasons for living appeared to be a central and important coping mechanism that allowed for engagement with the safety planning process despite internal obstacles.

## 4. Discussion

This research paper explores obstacles and solutions to safety plan use as identified over nine months by participants attending a three-week SPGI. The paper discusses two main themes: external obstacles and solutions, and internal obstacles and solutions.

Previous research examining barriers to treatment adherence continuously finds recurring obstacles for people with mental health challenges, particularly people experiencing suicidality. As identified by this research, there are links between the various sub-themes; the repetition observed among the various solutions across the two themes validates the complexity of the lived experience. Each obstacle is unlikely to act in isolation but likely interacts with and reinforces the others [25]. Exploring the complex relationships between each of the obstacles identified is beyond the scope of this analysis; however, through research, we know that complex socio-economic, cultural, environmental, and societal structures often interplay with one another, resulting in challenges to treatment and, in this instance, the practical application of safety plans. Rather than representing individual categories, the internal and external obstacles appear to interact. External stressors often had the potential to intensify experiences of emotional distress. Likewise, internal difficulties could reduce participants’ capacity to seek support or implement coping strategies. These findings suggest that for people with lived experience to engage with safety plans when needed, implementation requires consideration of both the individual’s internal psychological state and the broader environmental context in which crises occur.

The obstacles and solutions identified by participants aligned closely with the components of safety planning interventions. Participants described obstacles that interfered with recognising warning signs, engaging in internal coping strategies, accessing social and professional supports, and staying motivated to use safety plans during periods of distress. Consistent with previous research, these barriers reflected the emotional and environmental challenges often described during suicidal crises, highlighting the importance of collaboratively identifying practical strategies to overcome them [26,27]. Therefore, these findings show an important practical consideration for SPIs. Having a safety plan alone may be insufficient if individuals are unable to implement its strategies during periods of distress. Safety plans provide a structured framework for identifying coping strategies, sources of support, and making environments safer; as shown by the results of this research, their effectiveness can rely on an individual’s ability to access these strategies when experiencing intense, heightened emotional distress. The obstacles identified in this study suggest that using a safety plan may be influenced by factors both internal and externally experienced by the person. Future iterations of SPIs should therefore emphasise not only the development of personalised, co-produced safety plans, but also on anticipating potential barriers, identifying strategies, and practical steps that can increase the likelihood of plan use during periods of distress. A recurring and important protective factor which was identified across both themes was recognising personal and meaningful reasons for living, which was highlighted as a key way to address the challenges discussed. Within safety planning interventions, identifying meaningful reasons for living can strengthen an individual’s commitment to using their safety plan. This finding is consistent with research demonstrating that reasons for living function as an important protective factor against suicidal behaviour and may enhance engagement with safety planning strategies [27,28,29].

Participants also identified external barriers that could limit implementation of safety plans, including distressing environments, interpersonal conflict, unavailable support persons, work or academic pressures, substance use, social media exposure, and suicide bereavement. These findings reinforce the importance of collaboratively identifying perceived or anticipated obstacles during safety plan development, alongside realistic alternative coping strategies and support options. In particular, participants emphasised the value of identifying multiple support contacts rather than relying on one individual, consistent with recommendations within safety planning interventions. This finding aligns with broader suicidality literature identifying social support as a key protective factor, with increased access to supportive relationships and services associated with reduced suicide risk [30,31].

Internal obstacles were also described by participants. These were challenges that can impair the ability to use a safety plan during a suicidal crisis. Participants identified practical, peer-informed solutions to address these barriers, demonstrating the value of incorporating lived experience into safety planning interventions. It’s possible that the collaborative environment encouraged participants to develop personalised strategies that may increase the likelihood of safety plan utilisation during future crises. The participants led discussions which helped to overcome obstacles to safety plan implementation and collaboratively generated potential solutions. Although not directly examined in this study, this approach may have encouraged peer learning and collaborative problem-solving by providing opportunities for participants to share experiences and exchange practical strategies for implementing their safety plans. The group-based format of the intervention may have contributed uniquely to these findings. By bringing together individuals with lived experience of suicidality, participants were able to share experiences and discuss practical strategies for overcoming obstacles to help individuals implement their safety plans during times of distress. This allowed participants to listen to one another’s experiences and consequently broaden the range of coping strategies and support options available. Participants themselves identified solutions for one another’s obstacles rather than solely relying on a facilitator. This means that they were then perceived as realistic and acceptable. Recommendations described within the safety planning literature further support the relevance of these findings [6,7]. The participant-led identification of both obstacles and solutions represents a particular strength of this intervention. Through collaboration and identification of obstacles to safety plan use, participants identified strategies that were meaningful within their lived experience.

The implementation of safety plans, rather than their development alone, represents a critical challenge within suicide prevention. With the majority of research examining the development of safety plans, the greatest challenge is not necessarily constructing a safety plan but accessing and implementing it during periods of distress when cognitive flexibility and problem-solving are significantly impaired. Findings demonstrate that having a safety plan does not necessarily mean that it will be used during periods of acute distress. Rather, the effectiveness of safety planning depends not only on what is included in the plan, but also on supporting individuals to anticipate and overcome the practical obstacles that may arise when they need it most.

### 4.1. Clinical Implications

These findings have practical implications for clinicians implementing SPIs. Rather than focusing only on developing the steps and components of a safety plan with people who are experiencing suicidality, clinicians should explore potential obstacles that may prevent individuals from using their safety plan during periods of crisis or heightened distress. This includes discussing practical, emotional and environmental obstacles, as well as discussing the likelihood that these barriers may arise, and collaboratively identifying realistic coping strategies and sources of support. Reviews of safety plans with patients could also be beneficial, as barriers and available supports are likely to change over time. Therefore, through the incorporation of discussions of perceived or anticipated challenges into SPIs or clinician meetings with people experiencing suicide related distress, safety plans could end up becoming more feasible, in turn, making them more accessible to those who need them.

### 4.2. Limitations

There are several limitations in this study. The transferability of these findings should be considered. Participants were recruited from a single geographical region in Ireland and were predominantly White Irish, resulting in obstacles and solutions that may reflect the cultural, social, and the healthcare context in which the intervention was delivered. Experiences of safety plan use may differ across cultural groups, healthcare systems, and communities with varying levels of access to mental health services, social support, and suicide prevention resources. Participants were recruited from mental health services and were willing to engage in a group intervention. Therefore, the findings may not reflect the experiences of individuals who are not engaged with services, or who are unwilling or unable to participate in group interventions. Another methodological weakness of this research is that we don’t have data on how many participants shared the same obstacles, and likewise, how many participants shared the same solutions. Without this data, it’s difficult to assess the prevalence or consistency of obstacles or the effectiveness of specific solutions. A study constraint is the absence of individuals with lived experience in the deliveryof the intervention. Although perspectives from participants and practitioners provided valuable insights, the lack of involvement from a peer with lived experience may have limited the extent to which the intervention reflected the priorities and challenges faced by those who use safety plans. Another methodological consideration of the current study is the absence of direct participant quotations. Data were generated through a group discussion, where participants collaboratively identified barriers and solutions rather than providing individual accounts. As responses were captured as group-generated outputs rather than verbatim transcripts, direct quotations were not available. While this approach allowed for the identification of shared experiences across groups, it consequently limited the extent to which individual perspectives could be highlighted. A final constraint is that facilitators may have unintentionally been prompted to discuss barriers and solutions due to their prior experience delivering the intervention or the influence of social expectations.

### 4.3. Future Research Directions

Future research should seek to recruit more diverse and representative samples to improve the generalisability of findings. Consider collecting quantitative data alongside qualitative findings to determine the prevalence of specific barriers and facilitators to safety plan implementation. Likewise, future workr may benefit from adopting a co-production approach, where individuals with lived experience are involved in the design and evaluation of safety planning interventions. The participant-led discussions within this study highlight the value and necessity of involving lived experience perspectives in safety planning interventions. Furthering this involvement could help to develop more acceptable and, importantly, more meaningful support for individuals experiencing suicidality. Recommendations for future studiesshould include qualitative studies exploring participants’ experiences of using their safety plans after completing the intervention and identifying ongoing barriers and facilitators to implementation. Avenues for further investigation should also examine obstacles and solutions that may enhance the practical use of safety plans across different cultural healthcare settings to determine how transferable these findings are. Lastly, researchers should incorporate meaningful lived experience involvement through co-design approaches that may enhance the relevance and also the person-centred nature of safety planning interventions.

## 5. Conclusions

This study offers a novel contribution to the expanding body of literature on SPGIs. Continued investigation in this field is recommended. Moreover, further research into preventive strategies addressing the identified challenges is strongly encouraged.

## Figures and Tables

**Table 1:** Participant Demographics. (*n* = 43 participants).

Variable	*n*	%
Age (years)		
Mean (SD)	35.64 SD (13.95)	
Range	19–76	
Gender		
Male	14	33
Female	25	58
Non-binary	3	7
Identifies as male	1	2
Relationship status		
Single	25	58
Married	8	19
Divorced/separated	3	7
Widowed	1	2
Long-term relationship	6	14
Ethnicity		
White Irish	36	84
Other White background	7	16
Employment status		
Employed full-time	7	16
Employed part-time	9	21
Homemaker	2	5
Full-time student	6	14
On medical leave	9	21
Unemployed (<6 months, expects to work)	2	5
Unemployed (>6 months, but expects to work)	1	2
Unemployed (<6 months, does not expect to work)	1	2
Unemployed (>6 months, does not expect to work)	3	7
Laid off	1	2
Retired	2	5
Primary diagnosis		
Mood disorder	23	54
Anxiety disorder	11	26
Emotionally Unstable Personality Disorder (EUPD)	8	19
Substance misuse	1	2

**Table 2:** External Obstacles and Solutions.

Obstacle	Solutions
Time of Day/Week	Use calendar reminders to promote self-care before a difficult day/time Reward yourself for making safer choices Remembering that no time is a good or bad time of day Remembering to keep engaged in activities Listening to podcasts Catch yourself early- use your scale of intensity Book nice things to look forward to (e.g., a gig, holidays, tattoos)
Triggers	Remember 90 s feelings, e.g., remind yourself that feelings peak after 90 s. Change your environment; find new places or routes Building awareness of triggers: identifying and honouring them Try grounding strategies, e.g., cold water, a shower or a swim in the sea, eat a chilli
Person Not Being Available	If one person is unavailable, try the next person Practice more self-care Reframe the situation from an outside perspective Engage in comforting activities
Negative Interpersonal Relationships	Limit time with people who negatively impact you Use buffers or excuses to create space Set and maintain healthy boundaries; learn to say “‘no’” Identify who to avoid during times of distress
Distressing Environments	Avoid places that bring you down. Try different routes or different locations. Therapy Identify and prepare for triggers Spend time in spaces where you feel safe Spend time in communal areas Limitations in services
Work/Academic Stress	Find familiar and safe spaces Check in with yourself regularly Maintain work–life balance Seek financial or academic support Reward yourself for achievements Budget spending to reduce stress Learn to say “no” and outline your priorities Reach out to professionals or support systems Prioritise safety and basic needs
The Impact of Others Ending their Life	Talk to others; lean on social support Acknowledge the importance of grief Hold space for your feelings and needs
Substance Misuse	Seek support groups Talk to someone who understands Reduce or limit substance use Visualise future life Give excess to family and friends
Social media	Unfollow negative accounts/people who negatively impact you Limit time spent online (on certain apps) Avoid news and doom scrolling

**Table 3:** Internal Obstacles and Solutions.

Obstacle	Solutions
Executive functioning	Finding reasons to live (loved ones, memories, dreams, goals) Try a variety of things-Try! Try! Try! Practice routine and self-care regularly Thinking of what has worked before Set reminders for yourself Make photocopies of your safety plan and use the SafePlan app Do it now-while you feel OK Remind yourself that this will pass Find motivation-wanting to get better Make a plan Hold yourself accountable Keep a gratitude journal Practice new habits Keep copies of the safety plan in different places Share your safety plan with loved ones and people you trust
Lack of Motivation	Don’t do it alone- reach out for help when needed Remind yourself of your reasons for living (who/what motivates you), your loved ones, your goals, and dreams! Try and try again! Kobe mindset: “Do it tired”
Emotional Intensity	If one person is unavailable, try the next person Practice more self-care Reframe the situation from an outside perspective Engage in comforting activities
Self-Sabotage	Observation of your behaviours Remind yourself of your reasons for living Remember that your loved ones love you Prepare for difficult times Use affirmations Reminders from loved ones Create a code for loved ones to use if you need more support Write a letter to yourself when you are good, for all the reasons to stay alive Text a friend if you are struggling
Shame Related to Mental Health Difficulties	Look at photos of loved ones Think of what your safe spaces are; where is a place I can go to and feel safe Think of who your trusted and safe people are Think of your loved ones and of how much you are loved

**Table 4:** A Supplementary Coding Framework.

Theme	Subtheme	Example Codes
External Solution	Seek support groups	Intervention, therapy, post-vention, talk to someone
External Solution	Change your environment	Where is a safe place I can go, new places or routes, or go somewhere familiar (safe)
Internal Solution	Set reminders for yourself	Calendar reminders, preparation for those times, make a plan and hold yourself accountable

## Data Availability

The datasets generated and/or analysed during the study are not publicly available due to privacy regulations.
